# They’re Only Human! Tension and Stress Predict Performance of Softball Umpires in National Championships

**DOI:** 10.3390/sports13020035

**Published:** 2025-01-28

**Authors:** Ronald J. Houison, Andrea Lamont-Mills, Michael Kotiw, Peter C. Terry

**Affiliations:** 1 School of Psychology and Wellbeing, University of Southern Queensland, Toowoomba, QLD 4350, Australia; ron.houison@unisq.edu.au; 2 Academic Affairs Administration, University of Southern Queensland, Ipswich, QLD 4305, Australia; andrea.lamont-mills@unisq.edu.au; 3 Centre for Health Research, University of Southern Queensland, Springfield Central, QLD 4300, Australia; 4 School of Health and Medical Sciences, University of Southern Queensland, Ipswich, QLD 4305, Australia; mike.kotiw@unisq.edu.au

**Keywords:** BRUMS, mood profile, salivary cortisol, sports official

## Abstract

The psychology of sports officials is often overlooked in psychology research. The present study addressed this disparity by exploring relationships among the mood, stress, and performance of Australian softball umpires. Six male and two female participants aged 25–68 years (*M* = 48.95 ± 17.39 years) each completed the Brunel Mood Scale before games and provided saliva samples on multiple occasions prior to and after officiating games at two Australian National Softball Championships. Data from 65 games were analysed with performance assessed using Softball Australia’s umpire assessment tool. A significant positive relationship (*p* < 0.01) was found between tension scores and umpire performance. Using a stepwise regression analysis, tension scores and baseline cortisol level collectively explained 22.6% of the variance in umpire performance scores. These findings suggest that umpires require an optimal level of tension in the period leading up to competition to perform successfully, supporting the relationship between tension and performance first proposed by the inverted-U hypothesis.

## 1. Introduction

Umpires, referees, and judges (i.e., sports officials) are the least represented participants in team sports research despite performing a role equally critical to that of athletes [1]. In football (soccer) and American football, there are up to 22 players participating in the game at any one time, with up to seven officials supervising play [2,3]. In basketball, 10 players are supervised by up to three officials [4], and in elite-level baseball and softball, there are usually four to six officials managing games of 18 on-diamond players [5,6].

The role of the official in team sports is both complex and challenging due to a variety of co-occurring factors, including game speed, the number of participants, and the pressure to quickly make and communicate rulings [7,8]. Officials apply rules and maintain competitive fairness [9,10], apply their skills in complex situations, and adapt to changing game situations and competition intensity as their careers develop [10]. Officials are often subjected to derision [11], abuse, violence, and even death threats [12], and the pressure to perform competently can be stressful. Approximately 60% of Swedish football referees reported experiencing verbal abuse and 15% had reported experiencing physical aggression from participants or spectators [13]. Verbal abuse has been reported by 53.7% of English rugby union officials and 56.5% of English cricket umpires, and physical abuse by 6.4% and 10.0%, respectively [14]. Of direct relevance to the present study, 69% of softball umpires from the Missouri Amateur Softball Association reported experiencing verbal abuse and 6% reported physical abuse [15]. Changes in mood state can result from the individual’s performance [16] or mental health state [17], and stress levels can be affected by confrontation or commentary from players, coaches, or spectators [11] as a result of anticipating challenges in an upcoming game [18] or due to performance anxiety [19].

The challenging nature of the official’s role, the large number of sporting officials [20], the high performance expectations combined with the prevalence of criticism and abuse to which they are subjected point to the need for ongoing research to support the performance of officials. However, athletes and coaches have remained the primary focus of most psychological research in sport [21,22]. Although there has been an increase in officials-based research, Hancock et al. [9] recommended that more research is needed, particularly in the psychology of officials.

Moods and emotions are often viewed erroneously as synonymous. A mood has two main characteristics that distinguish it from an emotion: duration and intensity [23]. Moods typically are persistent and are of lower intensity than emotions. Also, unlike emotions, moods are generally experienced without any linked or triggering event [24], and are bi-dimensional in nature. That is, they vary on two continua from positive to negative assessment, and arousal levels range from deactivation to activation [25]. Self-report assessments, such as the Brunel Mood Scale (BRUMS) [26], are often used to quantify an individual’s mood scores [27]. Mood profiles, derived from six mood dimensions, tension, depression, anger, vigour, fatigue, and confusion [26], have been used in a variety of contexts, such as cardiac rehabilitation [28] and military personnel assessment [29]. However, the most common use of mood profiling is in the sporting context as a predictor of participant performance and an indicator of mental health risk [30,31].

Stress is the physiological and psychological response resulting from circumstances or events that are difficult to control or endure [32]. Such events activate the body’s stress systems [33,34]. The activation of these systems precipitates the secretion of hormones that are indicative of bodily stress: cortisol as a result of the body’s hypothalamic–pituitary–adrenal (HPA) axis [35] and adrenaline from sympathetic–adrenal–medullary system activation [34]. Adrenaline has a very short half-life of just minutes [34], making it impractical for use as a measure of stress in field-based research. Cortisol is regarded as a primary indicator of HPA axis activation [36], and the measurement of participant cortisol concentration in field work is often achieved using saliva samples. The cortisol concentration found in saliva has been reported to be proportional to that found in blood samples [34], and saliva samples can easily be provided without adding to the participants’ stress levels [37]. The most common way to analyse cortisol levels is by using immunoassay methods, such as enzyme-linked immunosorbent assay (ELISA), due to their convenience and reliability [38].

Stress has been shown to influence affective responses (including mood) and cortisol levels among both the general population and in sporting contexts. For example, a study of 120 participants who reported stressors and affect six times daily [39] demonstrated that both anticipating a stressor and experiencing a stressor increased salivary cortisol levels. Also, a study exploring the relationship between health problems, depression, and cortisol levels in a sample of 215 older adults found that depressive symptoms were associated with increased cortisol levels [40]. Further, Het and Wolf [41] found that participants treated with 30 mg of cortisol prior to undergoing the Trier Social Stress Test [42] reported mood states that were significantly less negative than participants treated with a placebo, suggesting that cortisol had a protective effect on mood. In an applied context involving participants in a program of guided musical imagery sessions, a decrease in cortisol concentration was shown to be significantly related to a decrease in mood disturbance [43]. In a sporting context, mood [17] and cortisol levels [44] have previously been shown to independently affect the performance of softball umpires.

With regard to assessing the combined effects of mood and cortisol, Filaire and associates [16] studied mood states, cortisol and testosterone concentrations, and the performance of 17 members of a French soccer team during a professional competition season. They reported a relationship between performance and mood state and proposed that combined psychological and physiological factors are of interest for monitoring stress in relation to performance in team sports. Filaire and associates [45] built upon this research with a study of the physiological and psychological states of 16 competitive tennis players. They reported a rise in cortisol concentration in anticipation of competition. Significant differences in cognitive anxiety and self-confidence scores between winners and losers were reported, which they noted was consistent with previous research by Terry et al. [46] and Chapman et al. [47]. Filaire et al. [16] recommended assessing the effects of psychological and physiological changes in combination when monitoring stress and performance, and this is the approach taken in the present study. The theoretical basis for the study was informed by the inverted-U hypothesis [48], which proposed a curvilinear relationship between tension and performance, and the model of mood and performance relationships in sports [49], which proposed that tension had a positive effect on performance except when depressive symptoms coexist.

Team sport environments are fast moving and have complex rules governing the interactions of many participants, creating what is often a heightened cognitive and/or physical load for officials [10]. Despite this, athletes as participants dominate sports performance research, with comparatively little research being published on the performance of sports officials [9]. Few papers have explored the interaction of mood and stress on the performance of sports officials and, in particular, among umpires of baseball and softball [50,51], which are sports played in many countries [52]. The present study sought to address this paucity of research by investigating relationships among mood, acute stress, and performance in Australian softball umpires.

Softball is a team sport and is similar to baseball. Several variants of the game exist, and the present study concentrated on the fast pitch variant. Two teams compete to hit a ball to advance players around the bases and score runs. The team scoring the most runs in a game is declared the winner [6]. The games are managed by umpires, of which there may be one–six appointed, depending on the level of competition. Commonly, there are at least four umpires appointed at elite levels of play [53]. The umpires officiate from various positions in the field of play and interact with the players while deciding the outcome of plays. One umpire, the “plate umpire”, is positioned behind home plate and is involved in most of the decisions made during the game. Other umpires can be found positioned initially at bases or in the outfield [53,54] and in this paper, they are termed “field umpires”. Field umpires are generally involved in adjudicating plays in their vicinity, and are under less stress than the plate umpire [44].

In this study, pre-game mood and baseline, and pre-game and post-game cortisol levels were measured among a sample of softball umpires to (a) assess patterns of mood and stress responses, and (b) determine if umpire performance could be predicted from mood and stress levels. The study methodology was similar in structure to the studies by Casanova et. al. [55] in which each participant provided multiple saliva samples and mood questionnaire responses over the course of a championship. It was hypothesised that there would be a significant increase in cortisol concentrations from baseline to pre-game (H1) and significant relationships among mood state, cortisol levels, and performance (H2).

## 2. Materials and Methods

### 2.1. Participants

Sixteen participants aged 25–68 years old (*M* = 52.27 ± 14.81 years), representing 76.2% of potential participants, were recruited from the 21 officials appointed to two of Softball Australia’s junior national softball championships. The sample included eleven male and five female participants (females *M_age_* = 40.20 ± 15.19 years, males *M_age_* = 52.27 ± 14.81 years). Seven participants attended the first championship, another seven participants attended the second championship, and a further two participants attended both championships. Participants’ accreditation levels ranged from Level 4 to 8 (*M* = 5.9 ± 0.9) with the higher-numbered level reflective of a more highly qualified umpire, and umpiring experience ranged from 7 to 35 years (*M* = 20.3 ± 8.2years). Participation was voluntary with no incentives provided. Data from eight participants aged 25–68 years old (*M* = 48.75 ± 18.78 years, 65 cases) were analysed following data screening. The final sample represented 38.1% of the population of interest and comprised six male and two female umpires (females *M_age_* = 27.0 ± 1.41 years, males *M_age_* = 55.67 ± 16.24 years).

#### 2.1.1. Ethics

Ethics approval #H19REA271 was granted by the University of Southern Queensland (UniSQ) Human Ethics Committee and approval #21BIO012A for the assay of biological material was granted by UniSQ Facilities Management. The recruitment of participants and written informed consent was obtained at umpire meetings the day prior to the commencement of each championship. Participants were allocated a unique ID based upon a randomly generated 5-digit number (random.org) to facilitate participant confidentiality.

#### 2.1.2. Data Screening

**Figure 1:**
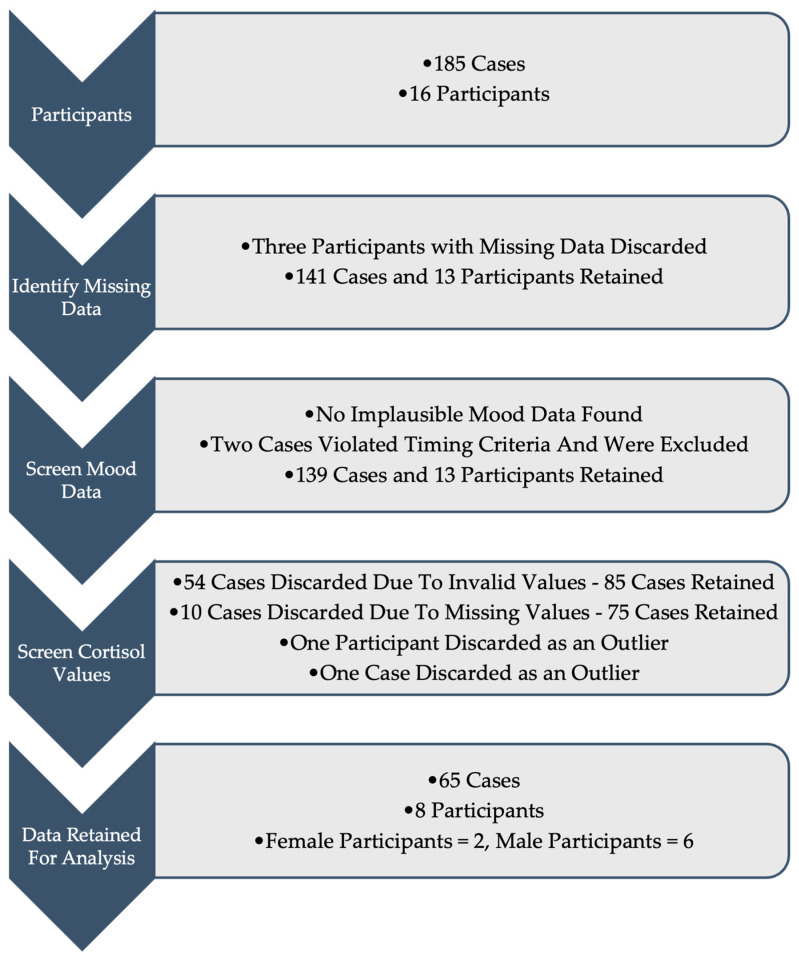
Data Screening Process.

Data provided by the 16 participants comprised 185 cases, one for each game officiated, which were carefully screened for inclusion in the analysis. Data were scrutinised in several steps (Figure 1). The first step was to identify missing data. Missing cortisol data reduced the number of participants from 16 to 13, leaving 141 cases. The next step was to screen the remaining cases for implausible or out-of-range data, and non-normal distributions. Mood data were screened for implausible response patterns, but none were found. However, two cases violated the timing criteria for completion of the mood scale (between 30 and 45 min prior to game commencement) and were removed from the dataset. Regarding non-normality among the mood data, significant deviation from univariate normality was expected given that the distribution of negative mood scores typically trends towards the lower end of the scale [56,57]. Positive skewness ranged from 1.46 to 8.12 for tension, depression, anger, and confusion scores. Kurtosis values were high, ranging from 2.27 to 66.0 for all subscales except vigour. After checking each case individually, no evidence of response bias was found [58,59] and therefore 139 cases were retained at this stage and no data transformations occurred.

The next step was to screen cortisol values for invalid concentration levels and missing values. Fifty-four cases were discarded due to invalid values, with cortisol concentration levels of ≤0 ng/mL, with 85 cases retained. Ten cases had missing pre-game and/or post-game values and were discarded, leaving 75 cases. One participant’s cortisol results had an abnormally large range, from 0.84 to 59.15 ng/mL, with a mean of 16.48 ng/mL, which was shown to be a statistical outlier and discarded, leaving 66 cases remaining. One further case was identified as an outlier and was discarded, and the remaining 65 cases from eight participants (female = 2, male = 6) went forward for analysis. Regarding umpire position on the diamond, 22 cases related to plate umpire (33.8% of cases) and 43 cases related to field umpire (66.2%). The number of games officiated by each participant is shown in Table 1. These 65 cases showed univariate normality with skewness = 0.31 (baseline), 0.88 (pre-game), and 1.18 (post-game), and kurtosis = −0.56 (baseline), 0.64 (pre-game), and 1.25 (post-game).

### 2.2. Measures

#### 2.2.1. Mood Assessment

Pre-game mood was measured using the BRUMS [26]. The questionnaire consists of 24 items of single-word mood descriptors with four items in each of the six subscales of anger (annoyed, bitter, angry, and bad tempered), confusion (confused, mixed up, muddled, and uncertain), depression (depressed, downhearted, unhappy, and miserable), fatigue (worn out, exhausted, sleepy, and tired), tension (panicky, anxious, worried, and nervous), and vigour (lively, energetic, active, and alert). A 5-point Likert scale ranging from 0 (not at all) to 4 (extremely) allowed respondents to indicate the strength of moods they were experiencing in response to the stem question, “How do you feel right now” [26]. Responses to each subscale item were totaled to give a subscale score ranging from 0 to 16 with higher scores indicating increased experience of the mood dimension being assessed [26]. BRUMS is psychometrically sound with multi-sample confirmatory factor analysis supporting the configural, metric, scalar, and residual invariance of the measurement model [56,57]. Cronbach alpha coefficients ranging from 0.74 to 0.90 for the BRUMS six subscales have been reported [56,57]. In the present study, Cronbach alpha coefficients ranged from 0.66 to 0.86 for the six subscales.

In this study, softball umpires were not considered to be athletes, as the role of the softball umpire is comparatively sedentary, unlike some other sports. Base and field umpires spend most of the game relatively stationary with occasional running up to 120 feet (36.6 m). The plate umpire is somewhat more active, squatting for every pitch thrown and running up to 60 feet (18.3 m) [53]. Hence, the mood scores of the participants were standardised using normative scores specific to male and female non-athletes [60]. Table 2 and Table 3 include the equivalent normative score (columns 2 to 7) for each raw score (column 1) across the six mood subscales.

#### 2.2.2. Umpire Performance Assessment

Softball Australia’s umpire assessment tool [61] was used to evaluate the performance of all championship umpires, including the participants, in every game per Softball Australia protocols [62]. This assessment process and tool were developed in the 1990s and are used to assess the performance of softball umpires in Australia, whether that be at national championships or as part of the process for candidates under examination for the next level of accreditation [61]. The assessment consists of five overall categories (General, Game Control, Judgement and Rules, Positioning and Calls, and Plate Work) scored under 23 performance sub-categories. Marks are assigned in each sub-category on a 5-point scale. The default score is 4 and participants are penalised for the first error observed and every second error thereafter in that sub-category (i.e., on the first, third, and fifth errors). A score of 5 is awarded only when no errors are attributed to the sub-category and the umpire’s performance in that sub-category is judged to have been of an exceptional standard. The umpire’s performance score is calculated firstly by totaling the sub-category scores in each category, and then the category scores are scaled using a conversion chart. The scaled scores are totaled to calculate the performance score. Higher scores signify better performance.

### 2.3. Procedure

This study was conducted at two championships held over two one-week periods in 2019 and 2020. Prior to the commencement of the championships Softball Australia invited all umpires appointed to officiate at the two championships, via email, to participate in the study. Participants provided written consent and demographic information including years of experience, accreditation level, age, and sex at umpire meetings held the day prior to each of the championships.

Participants were appointed to games as plate, base, or outfield umpires by the Umpire-in-Chief prior to the commencement of the championships. Variations to these appointments were made as needed during the championships by the Umpire-in-Chief. Base and outfield umpires are collectively termed “field umpires” in this study.

**Figure 2:**
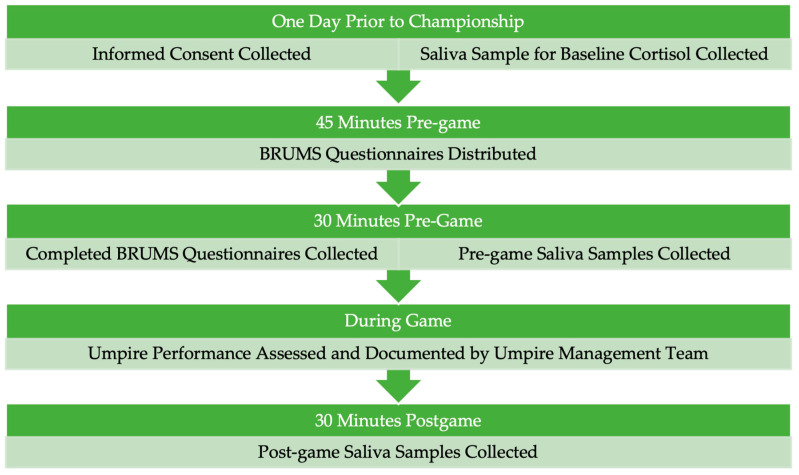
Data Collection Process and Timeline.

The methods and timelines involved in data collection were identical for each championship week (Figure 2). Participants’ informed consent and baseline salivary cortisol samples were collected at the pre-competition umpire meetings scheduled by the tournament Umpire-in-Chief. Baseline samples were provided at approximately 7:50 p.m. by participants officiating at one championship, and at approximately 3:50 p.m. by participants officiating at the other championship.

BRUMS data collection commenced approximately 45 min before each game when participants were issued a paper version of the scale [26]. This was completed by participants in the umpires’ change room and collected by the first author 30 min before game commencement. The questionnaire was completed no less than 20 min before the commencement of the second game when participants were appointed to consecutive games and the time between games was less than 60 min.

Pre-game and post-game saliva samples were collected by the first author approximately 30 min prior to the scheduled start of a game and 30 min after the completion of a game. Participants were directed not to eat, smoke, or drink (except for water), and not to brush their teeth inside 60 min before the baseline and pre-game saliva samples were collected, and during the 30 min before post-game samples were collected. Umpires appointed to consecutive games provided one sample between the two games with the single sample considered to be both a post-game and pre-game sample.

An alcove adjacent to the Umpire-in-Chief’s office on the lower level of the softball stadium provided participants with a relatively quiet and private environment in which to provide game-day saliva samples. Participants were requested not to smoke, eat, drink (except water), or brush their teeth in the 60 min prior to saliva samples being collected. Samples were collected in an unused paper cup and a cotton wool ball. The cotton ball was gently chewed by the participant until saturated with saliva and the participant then deposited the cotton ball into the paper cup. Saliva was squeezed from the cotton ball into the paper cup (samples of 50 microliters or more were deemed to be sufficient [63]) and transferred to a sterile micro centrifuge tubes with screw cap (Corning product number 403915), which was tagged with a unique number and logged in FreezerPro [64]. Collected samples were stored in a portable cooler on ice, then transferred to a domestic freezer at the end of each day and stored at −20 degrees Celsius. Participants officiated in between 7 and 13 games, and a total of 271 samples were collected, comprising 13 baseline samples, 131 pre-game samples, and 127 post-game samples.

After the championships, the samples were transferred on ice to the university campus for storage in the −80 degrees Celsius freezer pending assay. To ensure reliable cortisol data, every sample was assayed in duplicate (volume = of 12.5 µL) by qualified staff as recommended by the test manufacturer (Cortisol Competitive Human ELISA kit (EIAHCOR)) using commercial enzyme-linked immunosorbent assay (ELISA) kits with an analytical sensitivity = 17.3 pg/mL, assay range = 100–3200 pg/mL [63].

Every umpire’s in-game performance (including the participants) was assessed and documented on Excel spreadsheets in accordance with procedures specified by Softball Australia [62] by members of the umpire management team. The umpire assessment tool [61] was used to calculate the participants’ performance after the completion of the championships from the Excel spreadsheets, which were provided to the research team by Softball Australia’s Umpire-in-Chief (Development).

### 2.4. Statistical Analysis

All statistical analyses were conducted using SPSS for Mac, Version 29 [65]. Firstly, whether the diurnal variation of cortisol production would be a confounding variable was explored by calculating the correlation between the time of day that the sample was obtained and the pre-game cortisol level. Next, descriptive statistics for all participant mood scores were calculated and compared with population means using Welch’s *t*-tests, given that the present sample was much smaller than the normative sample. Descriptive statistics for cortisol concentrations at baseline, pre-game, and post-game were calculated, and compared using repeated-measures *t*-tests. Correlations among the six mood dimensions, three cortisol time samples, and umpire performance were calculated. A stepwise regression analysis was performed to predict umpiring performance from mood scores and cortisol concentrations. Effect sizes in the form of Cohen’s *d* were interpreted as small (0.20), moderate (0.50), or large (≥0.80), and correlation coefficients (*r*) as small (0.10), moderate (0.30), and large (≥0.50) [66].

## 3. Results

### 3.1. Time of Day and Cortisol Concentration

The secretion of cortisol is diurnal and, therefore, the time of data collection was a potential confounding variable [67,68]. A Pearson correlation coefficient was calculated to assess the relationship between the time of day the sample was provided and pre-game cortisol concentration. A very small correlation was reported between the two variables, r(64) = 0.08, *p* = 0.52. As only 0.64% of the variance in cortisol concentration was explained by the time of day that the sample was provided (Table 4), diurnal variation in cortisol secretion was not seen as a confounding factor.

### 3.2. Group Mood Profile

The collective mean mood profile for participants across all games was assessed. BRUMS raw scores were converted to standard scores (T-scores) [26] (Table 5). The participants as a group reported mean scores for tension, depression, anger, fatigue, and confusion that were significantly below population means, and scores for vigour that were above population means. Effect sizes were large for anger, confusion, depression, and fatigue; small to moderate for tension; and small for vigour. This indicates that, as a group, participants reported a mood profile associated with good performance and positive mental health.

### 3.3. Group Cortisol Concentration

The collective mean cortisol concentration for participants across all games was assessed with baseline to pre-game, baseline to post-game, and pre-game to post-game cortisol concentrations compared. As a group, participants recorded changes in mean cortisol concentration between baseline and pre-game (+84.4%), between pre- and post-game (−36.6%), and between baseline and post-game (+23.4%).

**Figure 3:**
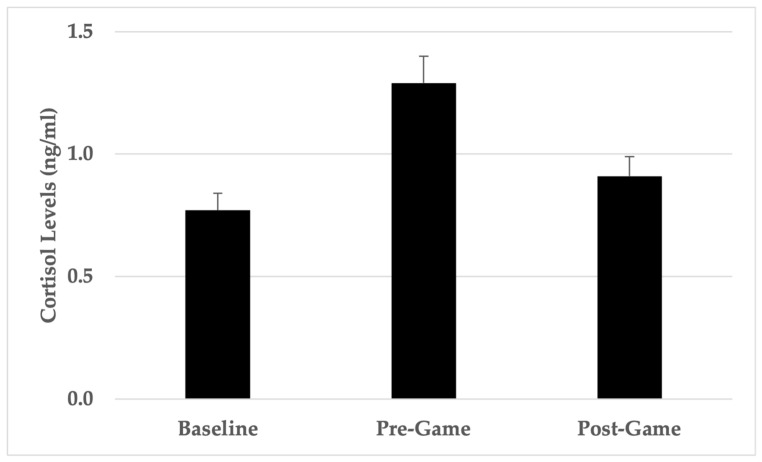
Group cortisol levels (mean ± standard error).

Repeated measures *t*-tests showed a significant increase in cortisol concentrations from baseline to pre-game, *t*(65) = −3.97, *p* < 0.001, *d* = −0.62 (moderate effect), and a significant decrease from pre-game to post-game, *t*(65) = 3.48, *p* < 0.001, *d* = 0.42 (moderate effect). No significant difference between baseline and post-game cortisol concentrations was reported, *t*(65) = −1.91, *p* = 0.06, *d* = −0.28 (small effect), (Figure 3).

Pearson correlations were computed to assess relationships between the six mood dimensions, the umpire-assessed score, and the three cortisol levels reported by participants for each game. A significant, moderate, positive correlation was reported between tension and assessed score, and between depression and anger. Also, significant, moderate, positive correlations were found between tension and cortisol levels at the three timepoints sampled (i.e., baseline, pre-game, and post-game. See Table 6).

A stepwise regression analysis was computed to determine predictor variables responsible for the variance in assessed score. Several assumptions underlying the use of regression analysis were checked. First, the distributional properties of the outcome variable, the assessed score, were within acceptable bounds (skewness = 0.80, kurtosis = −1.20) [66]. Second, there was no evidence of multicollinearity, with all inter-correlations among predictor variables at <0.7 (see Table 6) [66]. Third, standardised residuals were within acceptable bounds of +3.0 to −3.0, and Cook’s distances were also within the acceptable range of 0.0 to 1.0 [66]. However, the number of cases to predictor variables fell below the desirable ratio of 20:1, indicating that adjusted *R*^2^ should be used rather than the standard *R*^2^ to determine the percentage of explained variance [66]. Also, the linear relationships between the predictor variables (mood subscale scores and cortisol concentrations) and the outcome variable (assessed score) were, in most cases, apart from tension, below the recommended 0.3 threshold. Finally, although each case in the analysis related to a separate game, the same participants provided multiple mood and cortisol measures, breaching the assumption of independence. Therefore, given that some assumptions were not met, the generalisability of our findings to the larger population of softball umpires is limited [66]. Notwithstanding this limitation, results of the stepwise regression analysis indicated that tension and baseline cortisol level collectively explained 22.6% of the variance in the assessed score (see Table 7). No other predictor variables added significantly to the explained variance.

## 4. Discussion

Pre-game mood state and saliva samples were collected from softball umpires officiating at two Australian national championships to assess relationships between pre-game mood state, cortisol levels, and performance. Pre-game mood state data were collected and assessed similarly to previous studies of athletes [69] and the concentration of cortisol in the samples was collected and analysed similarly to studies of athletes [45,55,70], musicians [19], and academics [34]. As hypothesised, a significant rise in cortisol concentration from baseline to pre-game was found (H1) and significant relationships emerged between mood states, performance, and cortisol levels (H2), with the scores for tension and baseline cortisol level collectively explaining 22.6% of the variance in umpiring performance.

Cortisol levels vary throughout the day, peaking in the morning, with the lowest concentration between midnight and 3 am [67,68]. As pre-game saliva samples were obtained from participants throughout the day between 08:30 and 18:45, this variation had the potential to confound results. The non-significant correlation between cortisol concentration and time of sample, and the small (less than 1%) variance in the results explained by the diurnal variation in cortisol concentration lends support to the reliability of the stress findings.

The group mood profile showed the more negative dimensions of mood being below the population mean and the positive dimension (vigour) being above the population mean. This reflects an iceberg profile, which has been found to be associated with both good mental health and performance [71]. The small effect for vigour scores above the population mean was expected, as softball umpires are less physically active than officials in other team sports such as Australian Rules Football [72] and association football (soccer) [73]. Thus, high vigour is expected to be less important to softball umpiring performance than other mood factors. The decisions rendered by officials are sometimes controversial and are often at odds with the opinions of players and spectators, sometimes resulting in conflict [11,12]. Managing anger responses, especially when in conflict situations, is a critical skill for umpires. In the present sample, 98.5% of participants reported anger scores of zero, which has also been found to be associated with good performance and positive mental health [60].

Tension has been reported to increase cognitive load and thereby impact performance by affecting decision making [74]. Performance is thus expected to suffer if the official interprets tension as a negative emotional state [75]. In the present study, tension was significantly and positively correlated with performance. A conceptual model of mood and performance relationships in sports [49] suggests that when depressive symptoms are experienced, then tension has a negative effect on performance. However, in the absence of depressive symptoms, tension is expected to facilitate performance up to a point, although if tension continues to increase, reduced performance is expected, reflecting an inverted-U relationship [48]. It was anticipated that tension, being a stressor, would be reflected in cortisol concentration levels [16,39]. A weak, significant positive correlation was reported between tension and cortisol level at baseline, pre-game, and post-game. Pre-game cortisol levels were expected to rise, consistent with the findings of Filaire et al. [45], and to have a significant effect on performance, although it was found that baseline cortisol levels were more predictive of performance. Baseline cortisol concentration, in conjunction with tension, explained 22.6% of the variance in the assessed scores. This suggests that the mood state of umpires, especially their perceived tension, should be monitored leading into a national competition and maintained at an optimal level to facilitate performance. It should be noted that the combination of mood scores and cortisol values was a better predictor of umpiring performance that either mood [17] or cortisol [44] in isolation.

There are limitations associated with the present study. Firstly, the results were based on data derived from a small population base. Sports with larger accessible populations, for example baseball in the USA and association football (soccer) in Europe, would offer the opportunity to examine a greater range of parameters, such as the effect of the size of the crowd, the importance of the game, and any historical animosity between the teams. Although the present sample size was small, other studies with small sample sizes have also found stress and cortisol levels to be significantly related to performance [19,70]. The small sample size was reflective of several factors. Softball is a relatively small sport in Australia meaning smaller numbers of umpires are needed at national championships. A second limitation is that the collection of data was restricted to a single year due to national championships being cancelled the following year because of the COVID-19 pandemic. Nevertheless, significant results were reported with effects in the predicted direction. While moderate-to-large effects were found for the mood data, the effects were not as obvious in the cortisol data. Increasing the sample size will improve statistical power, allowing for the better detection of effects and improved generalisability of results. Future studies in the softball community should consider data collection across multiple events, both at national and state levels, to increase the number of cases collected and the number of participants. A third limitation relates to our use of multiple regression analysis when most, but not all, underlying assumptions were met. We addressed the effect of cases to predictor variables falling below the desirable ratio of 20:1 by using adjusted *R*^2^ to determine explained variance [66]. However, given that linear relationships between the predictor variables (mood subscale scores and cortisol concentrations) and the outcome variable (assessed score) were, in most cases, apart from tension, below the recommended 0.3 threshold, the results of the multiple regression should be treated with caution and preclude the generalisation of results to larger populations of umpires. However, it is posited that the findings in this study provide an impetus for further studies to examine the effects of mood and stress on the performance of officials in softball, baseball, and other sporting codes.

## 5. Conclusions

This study recruited Australian softball umpires officiating at junior national championships and examined the relationship between pre-game mood, cortisol levels at three timepoints, and umpire performance. The research aims were (a) to assess patterns of mood and stress responses, and (b) to determine if the performance of the umpires could be predicted from mood and stress levels. The hypotheses of a significant rise in pre-competition cortisol concentration and significant relationships between mood state, performance, and cortisol levels were supported.

The findings suggest that umpires require an optimal level of tension to perform at their best and future studies regarding the zone of optimal stress and mood state associated with optimum performance in sports other than softball should be encouraged. The results of this study are likely to promote interest and guide further study into umpire performance in countries with high levels of participation in softball and/or baseball.

## Figures and Tables

**Table 1:** Distribution of Cases (i.e., Games Officiated) by Participant.

Participant	Cases Analysed	Percent
20186	9	13.8
24294	8	12.3
37896	9	13.8
50451	11	16.9
53066	6	9.2
58995	7	10.8
71417	6	9.2
89243	9	13.8
	65	100

**Table 2:** Raw Scores and Equivalent Normative Scores for Male Non-athletes [60].

Raw Score	Mood Dimension T-Scores					
	Anger	Confusion	Depression	Fatigue	Tension	Vigour
0	43	42	44	37	43	30
1	46	45	47	40	45	33
2	50	49	50	42	48	35
3	53	52	53	45	51	38
4	56	55	56	47	54	41
5	59	59	59	50	57	43
6	62	62	63	53	60	46
7	66	65	66	55	64	48
8	69	68	69	58	67	51
9	72	72	72	60	70	54
10	75	75	75	63	73	56
11	79	78	79	65	76	59
12	82	82	82	68	79	62
13	85	85	85	70	82	64
14	88	88	88	73	86	67
15	91	91	91	75	89	69
16	95	95	95	78	91	72

**Table 3:** Raw Scores and Equivalent Normative Scores for Female Non-athletes [60].

Raw Score	Mood Dimension T-Scores					
	Anger	Confusion	Depression	Fatigue	Tension	Vigour
0	43	42	43	36	41	32
1	46	45	47	39	44	35
2	50	48	50	41	47	37
3	53	51	53	44	50	40
4	57	55	56	46	53	43
5	60	58	59	49	55	45
6	63	61	62	51	58	48
7	67	64	65	54	61	51
8	70	67	68	56	64	53
9	73	70	72	59	67	56
10	77	73	75	61	70	59
11	80	76	78	64	73	61
12	83	80	81	66	76	64
13	87	83	84	69	79	67
14	90	86	87	71	82	69
15	93	89	90	74	85	72
16	97	92	93	76	88	75

**Table 4:** Mean group cortisol levels (ng/mL) and time of day.

	*N*	Pre-Game Cortisol	Sample Time Range	Time of Day
Mean (SD)	65	1.29 (0.91)	08:30–18:45	14:16 (3.09)

Note. Based on data from 65 games. (SD) = standard deviation.

**Table 5:** Participant mood scores and comparison with non-athlete norms.

Dimension	Min	Max	Mean	SD	SE	*t*	*p*	*d*
Tension	43	67	47.71	5.26	0.65	−3.51	<0.001	−0.29
Depression	43	59	44.23	2.33	0.29	−19.96	<0.001	−0.79
Anger	43	46	43.05	0.37	0.05	−75.48	<0.001	−0.98
Vigour	33	72	52.29	8.65	1.07	2.14	0.036	0.24
Fatigue	36	63	40.20	4.82	0.60	−16.38	<0.001	−1.25
Confusion	42	49	42.29	1.11	0.14	−55.77	<0.001	−1.08

Note. Based on data from 65 games. SD = standard deviation, SE = standard error, *t* = *t*-test statistic, *p* = probability level, *d* = Cohen’s d (effect size).

**Table 6:** Correlation matrix of pre-game mood subscale scores and cortisol concentrations at baseline, pre-game, and post-game.

Measure	1	2	3	4	5	6	7	8	9	10
1. Tension	-									
2. Depression	−0.11	-								
3. Anger	−0.11	0.47 **	-							
4. Vigour	0.10	−0.07	−0.02	-						
5. Fatigue	−0.24	−0.01	−0.005	0.03	-					
6. Confusion	−0.14	−0.08	−0.03	−0.03	−0.07	-				
7. Assessed Score	0.41 **	−0.01	−0.11	0.16	−0.24	−0.04	-			
8. Baseline	0.30 *	−0.07	0.03	−0.24	−0.001	0.10	−0.15	-		
9. Pre-game	0.29 *	0.12	0.24	−0.07	−0.07	0.06	0.11	0.49 **	-	
10. Post-game	0.31 *	−0.05	0.02	−0.21	0.05	0.12	0.07	0.37 **	0.52 **	-

Note. Based on data from 65 games. ** Correlation is significant at the 0.01 level (2-tailed). * Correlation is significant at the 0.05 level (2-tailed).

**Table 7:** Stepwise regression analysis to predict assessed score from mood and cortisol (*N* = 65).

Measure	B	SE	*T*	*p*
Tension	0.88	0.20	3.06	<0.001
Baseline	−5.15	1.99	−2.59	0.012

Note: B = unstandardised Beta, SE = standard error, *t* = *t*-test statistic, *p* = probability level.

## Data Availability

Data will be uploaded to a repository prior to publication.
